# Five-Fraction Stereotactic Radiotherapy for Brain Metastases—A Retrospective Analysis

**DOI:** 10.3390/curroncol30020101

**Published:** 2023-01-17

**Authors:** Julian P. Layer, Katharina Layer, Gustavo R. Sarria, Fred Röhner, Cas S. Dejonckheere, Lea L. Friker, Thomas Zeyen, David Koch, Davide Scafa, Christina Leitzen, Mümtaz Köksal, Frederic Carsten Schmeel, Niklas Schäfer, Jennifer Landsberg, Michael Hölzel, Ulrich Herrlinger, Matthias Schneider, Frank A. Giordano, Leonard Christopher Schmeel

**Affiliations:** 1 Department of Radiation Oncology, University Hospital Bonn, University of Bonn, 53127 Bonn, Germany; 2 Institute of Experimental Oncology, University Hospital Bonn, University of Bonn, 53127 Bonn, Germany; 3 Institute of Neuropathology, University Hospital Bonn, University of Bonn, 53127 Bonn, Germany; 4 Division of Clinical Neuro-Oncology, Department of Neurology, University Hospital Bonn, 53127 Bonn, Germany; 5 Department of Neuroradiology, University Hospital Bonn, 53127 Bonn, Germany; 6 Department of Dermatology, University Hospital Bonn, 53127 Bonn, Germany; 7 Department of Neurosurgery, University Hospital Bonn, University of Bonn, 53127 Bonn, Germany; 8 Department of Radiation Oncology, University Medical Center Mannheim, University of Heidelberg, 68167 Mannheim, Germany

**Keywords:** FSRT, stereotactic radiotherapy, hypofractionation, brain metastases, radiation necrosis, toxicity

## Abstract

Purpose: To determine the safety and outcome profile of five-fraction stereotactic radiotherapy (FSRT) for brain metastases (BM), either as a definitive or adjuvant treatment. Methods: We assessed clinical data of patients receiving five fractions of 7 Gy each (cumulative physical dose of 35 Gy) to BM or surgical cavities. The primary endpoints were toxicity and radiation necrosis (RN) rates. Secondary endpoints were 1-year cumulative local control rate (LCR) and estimated overall survival (OS). Results: A total of 36 eligible patients receiving FSRT to a total of 49 targets were identified and included. The median follow up was 9 (1.1–56.2) months. The median age was 64.5 (34–92) years, the median ECOG score was 1, and the median Diagnostic-Specific Graded Prognostic Assessment (DS-GPA) score was 2. Treatment was well tolerated and there were no grade 3 adverse events or higher. The overall RN rate was 14.3% and the median time to RN was 12.9 (1.8–23.8) months. RN occurrence was associated with immunotherapy, young age (≤45 years), and large PTV. The cumulative 1-year local control rate was 83.1% and the estimated median local progression free-survival was 18.8 months. The estimated median overall survival was 11 (1.1–56.2) months and significantly superior in those patients presenting with RN. Conclusions: FSRT with 5 × 7 Gy represents a feasible, safe, and efficient fast track approach of intensified FSRT with acceptable LC and comparable RN rates for both the adjuvant and definitive RT settings.

## 1. Introduction

Brain metastases (BM) occur syn- or metachronously in up to 40% of patients with solid tumors [1,2]. Due to improved diagnostic imaging and prompt detection, but also novel systemic therapies and thus extended survival, prevalence is continuously increasing [2,3,4,5,6]. Even though overall survival (OS) does not only depend on BM [7,8], local treatment is indicated to prevent neurological impairment. Ablative options include surgery and radiotherapy (RT). While larger symptomatic lesions commonly require a priori resection and adjuvant radiotherapy [9], solitary stereotactic radiotherapy (SRT) is sufficient for multiple smaller and/or asymptomatic BM [10,11,12,13]. However, no standardized radiotherapy fractionation schema exists. Choosing an appropriate regimen depends on factors such as tumor localization, histology, and size [14,15]. Furthermore, dose prescription requires meticulous balance between desired local control and toxicity, such as radiation necrosis (RN) [16]. Due to its inferior toxicity profile, whole brain RT (WBRT) has been abandoned as a first-line strategy by most practitioners [11]. Standard SRT concepts either apply single-fraction stereotactic radiosurgery (SRS) or fractionated SRT (FSRT) with three [17] to twelve [18,19] fractions. Recent reports suggest the superior local control and reduced RN risk of FSRT [17,20] compared to SRS. Nonetheless, particularly in palliative situations, dose fractionation requires discretion to balance local control and side effects while avoiding potential overtreatment. Therefore, short-term treatment is generally preferred to improve quality of life (QOL) and limit patient visits and in-hospital time [21,22]. Among common FSRT concepts, five fractions of 5-6 Gy have been reported. The effectiveness and toxicity of 7 Gy single doses, comprising a slightly higher biologically effective dose and shortened treatment time, have not been well studied [23].

Even though initial prospective data suggest acceptable toxicity and comparable outcomes [24], this particular fractionation scheme appears somewhat outdated due to rather more protracted fractionation schemata. However, current RT techniques allow for both highly conformal planning and dose delivery, thereby possibly limiting toxicity [25]. Intensified hypofractionation strategies have subsequently gained reappraisal in the setting of oligometastatic solid tumors and are trending as the current treatment of choice for visceral and lymphatic metastases [26,27,28]. They are equally regarded as a minimal life disrupting approach in the principally curative setting [29].

The objective of this study was to report the safety and efficiency profile of using 5 × 7 Gy to treat BM in either an adjuvant or definitive setting.

## 2. Materials and Methods

### 2.1. Patient Selection

For this monocentric retrospective study, all consecutive patients with histologically confirmed solid tumors receiving either adjuvant or definitive FSRT with 5 × 7 Gy (cumulative physical dose 35 Gy, EQD2Gy = 49.6 Gy, BED = 59.5 Gy [α/β = 10 Gy]) to treat BM at University Hospital Bonn between 2016 and 2018 were assessed for eligibility. The inclusion criteria included age over 18 years, a pathology-confirmed malignant primary tumor, an ECOG score ≤ 2, and a total number of BM ≤ 10. The exclusion criteria were previous SRT of the same volume and simultaneous primary intracranial tumors.

### 2.2. Data Accrual

Clinical data were extracted from the clinical database and corresponding patient reports using SQL queries. The parameters of the treatments performed were extracted directly from the Eclipse planning system used for irradiation planning (Varian Eclipse 15.6, Varian Medical Systems, Palo Alto, CA, USA). Diagnostic-Specific Graded Prognostic Assessment (DS-GPA) [30] scores were calculated by standard procedures.

### 2.3. Fractionated Stereotactic Radiotherapy (FSRT)

Following interdisciplinary evaluation, linear accelerator-based FSRT was administered with intensity-modulated image-guided techniques, employing a 6 to 10 MV energy of 7-Gy single doses to a cumulative 35-Gy dose. The baseline magnetic resonance imaging (MRI) T1-Gd scan with 1 mm slice thickness was co-registered to planning computer tomography (CT) in all cases. The latter was acquired in a neutral supine position with patient fixation ensured by a thermoformed framed mask system. Gross tumor volume (GTV) was defined either as any T1-Gd contrast enhancing lesion or the resection cavity including any possible residual contrast enhancement. A 2 mm margin was added for the planning target volume (PTV) in both scenarios, as per institutional standards. Eclipse software was used for treatment planning and ExacTrac (Brainlab, München, Germany) was used for positioning matching. PTV coverage parameters included a D_min_ of 95% and a D_100%_ < 99% of the prescription dose. Organ at risk D_max_ constraints were defined as 22.5 Gy for optic nerves, 22.5 Gy for the optic chiasm, and 31.0 Gy for the brain stem.

### 2.4. Follow-Up

Follow-up (FU) visits included a physical examination and MRI imaging. Adverse events (AE) were assessed and graded by clinicians according to the National Cancer Institute Common Terminology Criteria for Adverse Events (CTCAE), Version 5.0 [31]. Acute toxicities were considered AEs occurring within the first three months of FU, whereas late toxicities were defined as all AEs recorded at a later timepoint. MRI reporting was performed according to the RANO criteria [32] by board-certified neuroradiologists. In case of doubt, in regard to either clinical or radiological response assessment, the interdisciplinary neuro-oncology tumor board was consulted. Uncertain cases received additional advanced imaging, including dynamic susceptibility contrast (DSC) MRI and MR spectroscopy. RN was diagnosed when any of the following conditions applied: (1) after initial suspected progressive disease (PD), at least two follow-up MR imaging time points showed no sign of PD; (2) advanced MRI incorporating DSC and diffusion weighted imaging (DWI) was suggestive of RN; (3) RN was confirmed pathologically after surgery.

### 2.5. Study Endpoints

The primary endpoints were RN and neurological adverse event rates according to the CTCAE criteria. Secondary endpoints included estimated OS and cumulative 1-year local control rates (LCRs). OS was defined as the time interval between the first day of SRT and the date of either the last FU (censoring) or death. LC was defined as an absence of MRI-radiographic progression in the previously irradiated metastatic volume. In case of re-resection and a pathologic confirmation of RN, this event was not considered a progression. Patients that were lost to FU or deceased prior to radiographic progression were censored at the last FU time point.

### 2.6. Statistical Analyses

Data analysis was performed with GraphPad Prism 9 (GraphPad Software, San Diego, CA, USA). If not stated otherwise, the Mann–Whitney test was employed to determine significance. The Chi-square test was used to assess the significance of contingency tables. For statistical comparison of high and low variable values, the collective was divided into the respective groups by its median. The Log-rank test was used for statistical assessment of survival and control rates and is presented according to the Kaplan–Meier method. Results with *p* < 0.05 were considered statistically significant. Specifically, statistical tests and analyses were performed as indicated in the respective figure legends. Figures were generated using GraphPad Prism 9 and Adobe Illustrator 2021 (Adobe Inc., Mountain View, CA, USA).

## 3. Results

### 3.1. Patient Characteristics

A total of 36 patients receiving FSRT to a total of 49 BM or resection cavities were screened and included. The median age was 64.5 (34–92) years, the median ECOG score was 1 (0–2), and the median DS-GPA score was 2 (0–4). The most frequent histology was non-small cell lung cancer (NSCLC; 33.3%), followed by melanoma (22.2%) and breast cancer (11.1%). Additionally, 9 of 36 (25%) patients had been treated with immunotherapy before RT and 24.5% had previously received RT, with five of these cases being WBRT. A total of six patients (16.7%) had received sequential SRS to distant lesions, three patients (8.3%) had received sequential FSRT, and five patients (13.9%) had received sequential WBRT. Further patient characteristics are depicted in Table 1. The most common BM location was the frontal lobe (28.6%). Regarding treatment setting, 30.6% received adjuvant and 69.4% definitive treatment. Median FU was 9 months (range 1.1 to 56.2 months). More detailed results can be found in Table 2.

### 3.2. Treatment and Dosimetry

**Figure 1:**
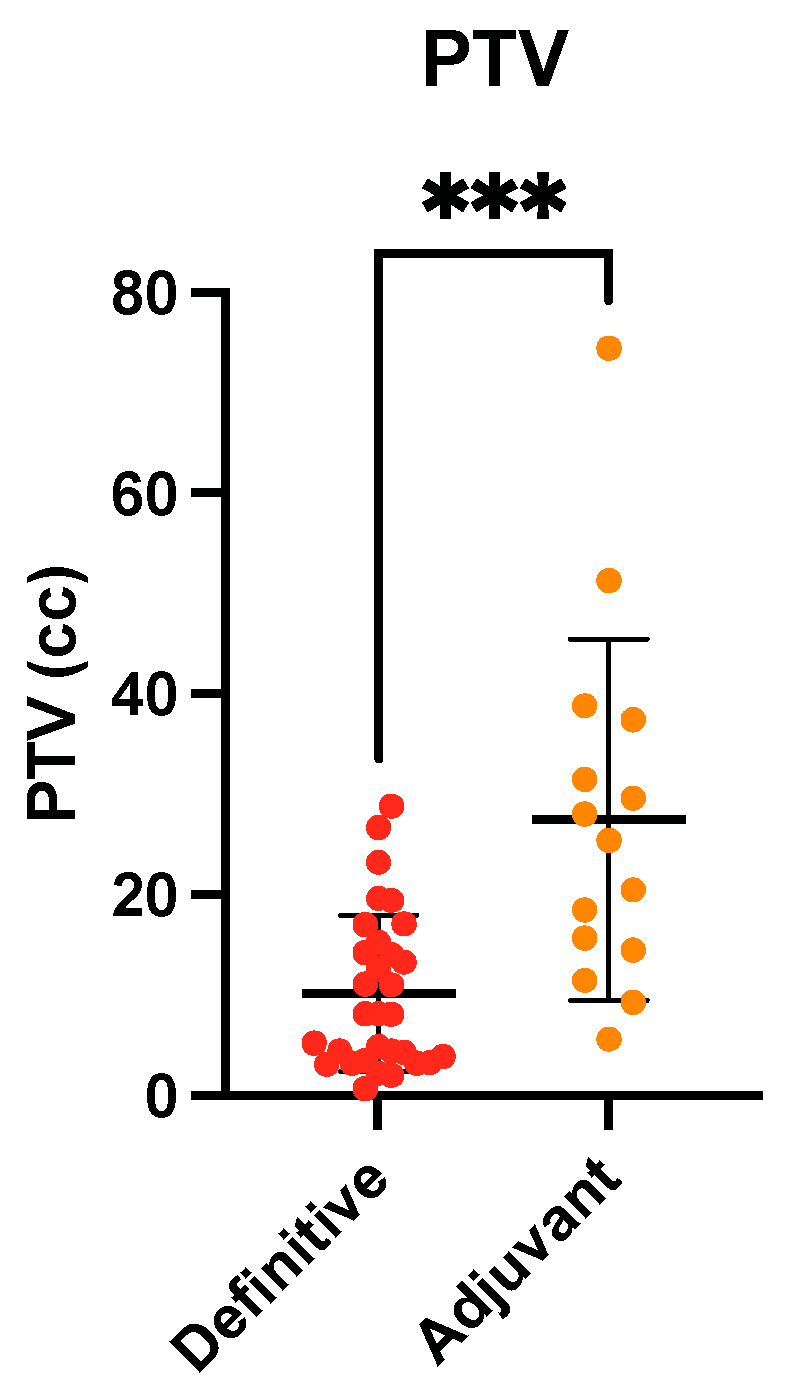
PTV of brain metastases undergoing definitive (red) vs. adjuvant (orange) SRT. *** *p* < 0.001, Mann–Whitney test. PTV: planning target volume; SRT: stereotactic radiotherapy.

All patients completed treatment. The median treatment time was 5 (5–8) days. The mean PTV_median_ dose was 35.8 (±1.28) Gy. The median PTV was 13.0 cc (0.7–74.4), with 8.2 cc (0.7–28.8) for definitive FSRT and 25.4 cc (5.6–74.4) for adjuvant treatment (*p* < 0.001). The PTV was significantly larger in the adjuvant RT subgroup than in the definitive RT subgroup (Figure 1; *p* < 0.001). A median of 2 (1–5) planning fields was used for RT planning. The median conformity index was 1.1 (0.2–3.5) and did not differ significantly between definitive and adjuvant SRT (*p* = 0.31). The median D_0.1cc_ was 37.4 Gy (36.0–42.1) and median brain V_50%_ was 40.9 cc (8.2–229.1). Further dosimetric features are described in Table 2 and Supplementary Figure S1.

### 3.3. Toxicity

A total of 54 events were recorded (Table 3). Of these, 44 were acute and 10 late adverse effects. In terms of grade, 75.9% were grade 1 and 24.1% grade 2 events. There were no grade 3 or higher adverse events (AE). The most common event was fatigue (30.6%), followed by cephalgia, nausea and vertigo (13.9%). The full list of AEs can be found in Table 4.

**Figure 2:**
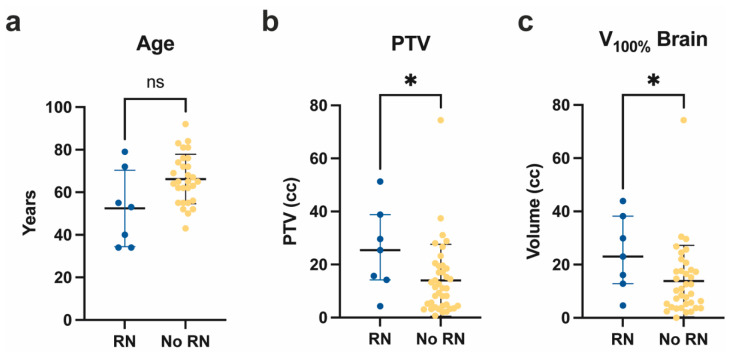
Characteristics of patients developing RN. Age (**a**), PTV of brain metastases (**b**), and brain V_100%_ (**c**) of patients developing (blue) vs. not developing (yellow) RN after SRT with 5 × 7 Gy. * *p* < 0.05; ns: not significant (*p* ≥ 0.05); Mann–Whitney test. PTV: planning target volume; RN: radiation necrosis; SRT: stereotactic radiotherapy.

RN was observed in seven targets (14.3%). Of these, one was pathologically confirmed. No patient had symptomatic RN ≥ grade 3. Four RN cases were located in the parietal lobe. Thus, 57.1% of patients with parietal metastatic localization developed RN during FU. The other RN cases occurred in the frontal, temporal, and occipital lobes. The tumor histology of RN patients was melanoma in three patients (RN incidence 37.5%), lung cancer (16.7%) in two patients, and breast and renal cancer in one patient. Median time to RN was 387 (53–726) days. Additionally, 57.1% of patients with RN had received immunotherapy (IT) prior to RT, and 71.4% of the patients had received SRT as an adjuvant treatment. A total of 75% of the RN patients receiving IT suffered from severe associated immunologic side effects (such as pancreatitis or hypophysitis) that eventually caused treatment interruption. Further characteristics of the RN patients can be found in Supplementary Table S1. At 53 (34–79) years, the median age of patients with RN was lower compared to the non-RN cohort (*p* = 0.05; Figure 2a). Patients ≤ 45 years of age harbored a significantly higher RN risk (*p* = 0.0003). The PTV significantly correlated with RN (25.4 vs. 11.1 cc; *p* = 0.04, Figure 2b). PTV D_0.1cc_ was significantly lower in patients developing RN (*p* = 0.03), while conformity index, V_10Gy_, and V_20Gy_ were not significantly different (*p* > 0.05). V_100%_ of the brain was significantly larger in RN developing lesions (*p* = 0.04; Figure 2c).

### 3.4. Survival and Control Outcomes

**Figure 3:**
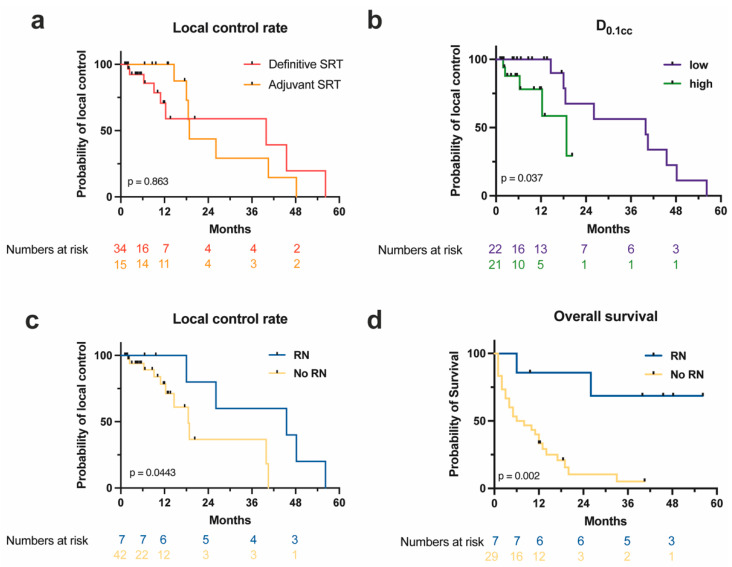
Local control and survival depending on patient characteristics. (**a**) Local control rate over time in months for definitive (red) and adjuvant (orange) SRT with 5 × 7 Gy (Log-rank test). (**b**) Local control rate over time in months for low (purple) vs. high (green) PTV D_0.1cc_ in FSRT with 5 × 7 Gy (Log-rank test). (**c**,**d**) Local control rate (**c**) and overall survival (**d**) over time in months of patients developing (blue) vs. not developing (yellow) RN after SRT with 5 × 7 Gy (Log-rank test). PTV: planning target volume; RN: radiation necrosis; SRT: stereotactic radiotherapy.

The cumulative 1-year local control rate (LCR) was 83.1%, while the 2-year LCR was 50%. After 3 years, the LCR was 41.7%. The median PFS was 18.8 months. The 1-year LCR was 100% for adjuvant RT vs. 70.8% for definitive RT. However, this control benefit for adjuvant RT was not significant (Figure 3a; *p* = 0.86), as it was restricted to the first 18 months of FU. The 2-year LCR was 43.8% in the adjuvant RT subgroup vs. 59% for the definitive RT subgroup. While local control was superior in the subgroup of FSRT with smaller PTV, this difference was not significant. There was no significant difference for LC in brain V_100%_ and V_50%_, but patients with lower PTV D_0.1cc_ had a significantly higher LCR (Figure 3b; *p* = 0.037). RN was significantly associated with better local control (*p* = 0.04; Figure 3c). Median local PFS for the subgroup of patients developing RN was 45.5 months, compared to 18.5 months for patients that did not develop RN.

Only one patient (2.8%) died due to radiology-confirmed local intracerebral progression, whereas 19.4% died of systemic progression. In addition, 53.1% of patients developed distant cerebral metastases and 63.3% progressed systemically, as confirmed by radiologic FU imaging. Median OS was 11 months, and OS was significantly superior in the RN subgroup (39.9 months) compared to the non-RN subgroup (7.0 months) (Figure 3d; *p* = 0.023).

## 4. Discussion

Interdisciplinary treatment of BM remains challenging and requires individualized shared decision making by the patients and their involved specialists. Given a continuously aging population with longer life expectancy, improved diagnostic tools, technical progress, and the rise of targeted therapies, the incidence of BM is likely to continue to increase [2,6]. However, similar to patients in our series, patient mortality is mostly due to systemic progression rather than local intracerebral progression [7]. Therefore, achieving and maintaining local control of brain metastases through RT is of utmost importance for symptom control and improved quality of life [33], despite the fact that it may not relevantly prolong OS [34]. Achieving sufficient local control and avoiding excessive toxicity requires careful therapeutic balancing. While single-fraction SRS has been widely adopted and likely has the highest biological effective dose (BED), either the location or volume of BM may necessitate fractionated alternatives [17,20]. Larger brain metastases were shown to benefit from FSRT with a relative reduction in RN rate, yielding a similar or even better LCR than SRS [35].

A systematic meta-analysis of eleven studies reporting a 6-month 80% LCR after SRT for BM recommended, in referral to a single fraction of 20 Gy, a minimum EQD2 dose of 40 Gy using an α/β of 12 Gy [36]. However, among the various available options, optimal SRT dosing and fractionation for BM remains controversial with no general consensus. Emerging evidence suggests that FSRT for BM improves patients’ QOL [11] and leads to both treatment and in-patient times being as short as possible [21,22]. This is of particular importance in BM patients with dismal prognosis and neurological deficits impairing individual mobility. For the same reasons, WBRT in BM patients ineligible for SRT has been questioned by the results of the QUARTZ trial [37]. Besides patient-centered beneficial aspects, intensified hypofractionation may also translate into socioeconomic cost reductions [38,39,40] while potentially allowing increased patient numbers and timely treatment access. The ongoing COVID-19 pandemic, with dynamically shifting and hardly predictable short-term patient numbers [41], has revealed the necessity of optimizing resources and time with the aim of reducing unnecessary harmful patient exposure to healthcare facilities [42]. This is the first detailed report on a fast track five-fraction SRT approach covering toxicity and, particularly, RN rate while also including dosimetry and outcome parameters.

Our data provide preliminary evidence on five-fraction SRT as a safe and feasible approach with both optimal adjuvant and definitive outcomes. Previously reported LCRs vary significantly and merely a handful of series have reported on this (Table 5) [24,43,44,45,46]. Our results resemble those cited above. Nevertheless, in comparison to other fractionation schemes (Table 6) [12,16,17,18,47,48,49,50,51,52,53,54,55,56] and intraoperative RT (IORT) [57], the LCR appears slightly weaker in our series, although it yields reasonable overall local control. This may be due to the specific composition of the patient cohort included. While melanoma was relatively overrepresented in our collective (22.2%), levels of NSCLC (33.3%) and breast cancer (11.1%) were below the average. The median age of 64.5 years was relatively high compared to other studies and the median GPA of 2 was relatively low. This rather unfavorable prognostic setting might have led to lower OS and a relevant number of lost-to-FU cases, which partially explains the pronounced drop in the 2-year LCR. The crossover in the LCR between definitive and adjuvant RT in the second year of FU may be explained by the significantly larger PTVs treated in the adjuvant setting, which might also explain the improved LCR with lower D_0.1cc_ as larger PTVs require higher central doses.

Notably, no grade 3 AEs occurred under or after FSRT. Compacted treatment time and dose escalation were not associated with a higher risk of AEs compared to more commonly applied schemes. This holds not only true for acute toxicity and late toxicity, but also for the anticipated RN risk. Even though SRS was associated with a higher risk of RN in previous series [16], we did not observe an increased RN rate or any grade 3 or higher clinical appearance in this patient collective with intensified hypofractionation. While the heterogeneity of our collective in terms of tumor entities and treatment parameters may contribute to RN development, the RN rate reported is in line with several other studies on other fractionation schemes that reported rates of up to 18% [54] in the definitive setting and up to 21.1% [51] in the adjuvant setting (Table 6). Similar five-fraction studies by Jeong et al. and Lischalk et al. previously reported RN rates of 15.8% [44] and 20% [53], respectively. Of note, 50% of these latter patients had received a prescribed total dose of 40 Gy and both RN patients requiring surgery had also received a maximum dose of more than 41 Gy, whereas in this collective only three lesions (6.1%) reached this dose level. Comparable with our results, an increased risk of RN with increasing tumor volumes has been reported in previous studies [16,49,51,54]. Additionally, the time to occurrence of RN is in line with the literature [50], though there is inconsistent data regarding the anatomic brain regions at particular risk of RN. Accordingly, despite there being reports of high RN incidence in the parietal lobe [12], others have conflictingly reported on increased RN rates in different brain sections [48,58]. Increased RN rates among melanoma BM are most likely due to more frequently applied IT, which was previously identified as an independent risk factor for RN [44]. As patients receiving targeted and pro-immunogenic therapies were underrepresented in earlier preceding studies, the comparable RN rate described here is even more compelling. Notably, 75% of the RN patients receiving IT in this study discontinued treatment due to intolerable immunologic side effects. Larger PTV size and lower D_0.1cc_ were associated with RN, while other dosimetric parameters were not related to either RN or LCR. This implies that immune therapy and lesion volume are relatively more relevant than other dosimetric factors for RN. While the longer OS of younger patients may contribute to the higher incidence of RN observed in this series, the contribution of immunological factors cannot be ruled out. Even though the exact mechanisms remain unknown, the link between irradiation and vascular or glial damage promoting a proinflammatory tumor microenvironment (TME) with subsequent activation of microglia, macrophages, and CD3+ T-cells has been established [59,60]. FSRT was shown to induce tumor immunogenicity [61,62]; furthermore, RN after SRS seems to be associated with improved OS [52]. Favoring an immunologically ‘hot’ TME may induce both favorable and unfavorable side effects, such as tumoricidal immune cell activity and RN. Therefore, despite an overall good clinical outcome, careful patient selection and close FU is suggested in order to avoid increased toxicity.

Although this study is the most comprehensive manuscript to date reporting on 5 × 7 Gy FSRT, it carries several limitations. Data were collected retrospectively from a rather heterogeneous patient collective undergoing adjuvant and definitive RT for several different entities. The fractionation regimen was at the physicians’ discretion, and for most patients the decision between FSRT and SRS was based on lesion volumes. Other reasons for FSRT included glucocorticoid intolerance of any kind (i.e., allergic reactions, gastric or duodenal ulcers, glaucoma, uncontrolled diabetes, sleep disturbances), which were routinely administered preceding SRS in our clinic during the study period. Low average performance and prognostic scores caused detriments in FU and PFS, and thus rather poor OS outcomes. Taken together, patient selection bias might have led to discordant outcomes when compared to the available literature. However, this collective represents an average patient population at a German university hospital and reflects a real-world situation instead of a carefully selected study population. Prospective randomized trials are needed to bring further insight into the optimal fractionation scheme for BM. The first randomized controlled phase III trial investigating SRS vs. FSRT for BM with a diameter of 2 cm to 4 cm was recently initiated [19]. Further studies that incorporate accelerated, intensified hypofractionated fractionation schemes are warranted. Additionally, the best treatment sequencing has not been defined yet. Recently, an NRG study protocol initiated recruiting, seeking to determine the best sequencing between pre- or post-operative SRS for BM (NCT05438212). The impact of short-term interventions on QOL will be the main focus of future developments in FSRT.

## 5. Conclusions

Five fractions of 7 Gy each appears to be a safe and effective alternative to more protracted fractionation schemes. Intensified FSRT might yield acceptable local control and toxicity rates in both the adjuvant and definitive RT settings while also maintaining short treatment times. These hypothesis-generating findings should be further studied within a clinical trial.

## Figures and Tables

**Table 1:** Patient characteristics.

Patient Characteristic	*n* (%)	Median (Range)/Mean (±SD)
Total number	36 (100)	
Male	20 (55.6)	
Female	16 (44.4)	
Age (years)		64.5 (34–92)
Total number of brain lesions		3 (1–10)
ECOG performance score		1 (0–2)
0	16 (44.4)	
1	11 (30.6)	
2	9 (25)	
DS-GPA		2 (0–4)/2.1 (±0.98)
Histology		
NSCLC	12 (33.3)	
Melanoma	8 (22.2)	
Breast	4 (11.1)	
SCLC	3 (8.3)	
CRC	2 (5.6)	
Esophageal	1 (2.8)	
Pancreatic	1 (2.8)	
Thyroid	1 (2.8)	
Ovarian	1 (2.8)	
SCC	1 (2.8)	
RCC	1 (2.8)	
Sarcoma	1 (2.8)	
Immunotherapy		
Yes	9 (25)	
No	27 (75)	
Previous RT		
Yes	7 (19.4)	
No	29 (80.6)	
Sequential RT to distant lesions		
SRS	6 (16.7)	
FSRT	3 (8.3)	
WBRT	5 (13.9	

CRC: colorectal cancer; FSRT: fractionated stereotactic radiotherapy; NSCLC: non-small cell lung cancer; RCC: renal cell carcinoma; RT: radiotherapy; SCC: squamous cell carcinoma; SCLC: small cell lung cancer; SD: standard deviation, SRS: stereotactic radiosurgery; WBRT: whole brain radiotherapy.

**Table 2:** Lesion characteristics.

Lesion Characteristic	*n* (%)	Median (Range)/Mean (±SD)
Total number	49 (100)	
Location		
Frontal	14 (28.6)	
Occipital	9 (18.4)	
Cerebellum	9 (18.4)	
Temporal	7 (14.3)	
Parietal	7 (14.3)	
Central	3 (6.1)	
Treatment setting		
Definitive	34 (69.4)	
Adjuvant	15 (30.6)	
Immunotherapy		
Yes	12 (24.5)	
No	37 (75.5)	
Previous RT		
Yes	12 (24.5)	
No	37 (75.5)	
Radiation necrosis		
Yes	7 (14.3)	
No	42 (85.3)	
PTV (cc)		13 (0.7–74.4)/15.8 (±14.4)
Conformity index		1.06 (0.21–3.5)/1.14 (±0.43)

PTV: planning target volume; RT: radiotherapy.

**Table 3:** Adverse events overview.

Grade	Acute Toxicity	Late Toxicity	Total
Grade 1	33 (61.1%)	7 (13%)	40 (74.1%)
Grade 2	11 (20.3%)	3 (5.6%)	14 (25.9%)
Grade 3+	0	0	0
Total	44 (81.4%)	10 (18.6%)	54 (100%)

**Table 4:** Full list of adverse events by grade.

Adverse Event	Grade 1	Grade 2	Grade 3+	Total
Fatigue	5	6	0	11 (30.6%)
Cephalgia	7	2	0	5 (13.9%)
Vertigo	4	1	0	5 (13.9%)
Nausea	5	0	0	5 (13.9%)
Alopecia	3	1	0	4 (11.1%)
Neuropathies	1	1	0	2 (5.6%)
Cognitive deterioration	2	0	0	2 (5.6%)
Fever	2	0	0	2 (5.6%)
Gait deterioration	2	0	0	2 (5.6%)
Skin reactions	2	0	0	2 (5.6%)
Mucositis	0	1	0	1 (2.8%)
Dysphagia	0	1	0	1 (2.8%)
Seizures	0	1	0	1 (2.8%)
Anemia	1	0	0	1 (2.8%)
Ataxia	1	0	0	1 (2.8%)
Cramps	1	0	0	1 (2.8%)
Gastrointestinal	1	0	0	1 (2.8%)
Tremor	1	0	0	1 (2.8%)
Viscerocranial pain	1	0	0	1 (2.8%)
Visional impairment	1	0	0	1 (2.8%)
	40	14	0	54 (100%)

**Table 5:** Studies on the FSRT of brain metastases with 5 × 7 Gy.

Authors	Year	Dose (Gy)	RT Setting	BM Size (Median)	RT Technique	Lesions	Histology	1y-LCR (%)	2y-LCR (%)	RN Rate (%)	Median Time to RN (Months)	Toxicity
Current series	2022	7/35	both	13 cc	Conventional FSRT	49	mixed	83.1	50	14.3	12.7	0% G3+
Di Perri et. al. [43]	2020	7/35	both	11 cc	Cyberknife FSRT	89	mixed	62.5	n. a.	>40	n. a.	n. a.
Ernst-Stecken et al. [24]	2006	7/35	definitive	13 cc	Conventional FSRT	72	mixed	76	n. a.	n. a.	n. a.	2% G3+
Jeong et al. [44]	2015	7/35	definitive	17.6 cc	Cyberknife FSRT	38	mixed	87	65.2	15.8	10.5	n. a.
Koide et al. [45]	2019	7/35	definitive	7.2 cc	Conventional FSRT	58	mixed	64.7	n. a.	3.5	n. a.	0% G3+
Mengue et al. [46]	2020	7/35	both	2.3 cm	Cyberknife FSRT	158	mixed	<80	<60	NA	n. a.	n. a.

BM: brain metastases; FSRT: fractionated stereotactic radiotherapy; LCR: local control rate; NA: not available; n. a.: not assessed; RN: radiation necrosis; RT: radiotherapy.

**Table 6:** Studies on the FSRT of brain metastases with other fractionation schemes.

Authors	Year	Dose (Gy)	RT Setting	BM Size (Median)	RT Technique	Lesions	Histology	1y-LCR (%)	2y-LCR (%)	RN Rate (%)	Median Time to RN (Months)	Toxicity
Brown et al. [47]	2017	12–20	adjuvant	<3 cm	Conventional SRS	93	mixed	61.8	n. a.	n. a.	n. a.	39% G3+
Choi et al. [48]	2021	Median 20	definitive	0.5 cc	Conventional SRS	311	melanoma	n. a.	n. a.	6.1	10.2	n. a.
Doré et al. [49]	2017	7.7/23.3	adjuvant	11.4 cc	Conventional FSRT	95	mixed	84	n. a.	20.6	15	n. a.
Eitz et al. [50]	2020	Median 6/30	adjuvant	23.9 cc	Conventional FSRT	581	mixed	84	75	8.6	13.1	6.9% G3
Fokas et al. [18]	2012	5/35	both	2 cc	Conventional FSRT	61	mixed	75	n. a.	1.6	n. a.	2% G3+
Jhaveri et al. [51]	2019	5–7/21–35	adjuvant	15/20 cc	Conventional FSRT	139	mixed	84.8	n. a.	21.1	n. a.	n. a.
Kohutek et al. [16]	2015	15–22	definitive	1.1 cm	Conventional SRS	271	mixed	n. a.	n. a.	25.8	10.7	n. a.
Lehrer et al. [52]	2022	Median 20	definitive	1.6 cc	Conventional SRS	4,536	mixed	90.5	n. a.	9.8	n. a.	n. a.
Lischalk et al. [53]	2015	6–8/30–40	definitive	5.6 cc	Cyberknife FSRT	13	NSCLC	90	90	15.4	11	15% G3+
Minniti et al. [12]	2011	15–20	definitive	1.9 cc	Conventional SRS	310	mixed	92	84	24	11	5.8% G3+
Minniti et al. [54]	2014	9–12/27–36	definitive	16.4 cc	Conventional FSRT	171	mixed	88	72	18	12	4% G3+
Minniti et al. [17]	2016	9/27	definitive	17.9 cc	Conventional FSRT	138	mixed	91	n. a.	8	12	n. a.
	2016	15–18	definitive	12.2 cc	Conventional SRS	151	mixed	77	n. a.	20	10	n. a.
Piras et al. [55]	2022	6–8/30–40	definitive	1.8 cc	Conventional FSRT	57	mixed	n. a.	n. a.	2.4	9	7% G2 0% G3
Wegner et al. [56]	2015	8/24	definitive	15.6 cc	Conventional FSRT	36	mixed	63	n. a.	0	n. d.	0% G3

BM: brain metastases; FSRT: fractionated stereotactic radiotherapy; LCR: local control rate; NA: not available; n. a.: not assessed; NSCLC: non-small cell lung cancer; RN: radiation necrosis; RT: radiotherapy; SRS: stereotactic radiosurgery.

## Data Availability

The data presented in this study are available in this article (and Supplementary Materials).

## Supplementary Materials

The following supporting information can be downloaded at: https://www.mdpi.com/article/10.3390/curroncol30020101/s1, Figure S1: Dosimetric specifications of patients; Table S1: Characteristics of patients with radiation necrosis.
