# Supplementary material for: Five-Fraction Stereotactic Radiotherapy for Brain Metastases—A Retrospective Analysis
Source: Curr Oncol. 2023 Jan 17;30(2):1300–13. doi: 10.3390/curroncol30020101 (PMC9955428; doi:10.3390/curroncol30020101)
Supplement: Supplementary file 1 [file curroncol-30-00101-s001.zip › Supplementary Layer JP et al/Suppl. Tables Layer JP et al.pdf]

Suppl. Table S1: Characteristics of patients with radiation necrosis.

Patient ID	Histology	Age (years)	KPS	Location	Treatment setting	Previous RT	Number of BMs	ITX	ITX discontinuation	PTV (cc)	Time to RN (days)	PFS (months)	OS (months)
32	Melanoma	40	100	Parietal	Definitive	No	1	Anti-PD1	Crohn reactivation	4.3	726	45.5	45.5
29	NSCLC	55	90	Occipital	Definitive	3 x 9 Gy contralat	2	No	-	14.2	675	56.2	56.2
7	Breast	34	90	Parietal	Adjuvant	No	3	No	-	51.3	546	18	39.9
2	Melanoma	34	100	Frontal	Adjuvant	No	1	Anti-CTLA4/ PD-1	Pancreatitis	15.7	387	48.2	48.2
15	SCLC	53	80	Parietal	Adjuvant	No	1	No	-	25.4	290	9.6	9.6
23	RCC	72	60	Parietal	Adjuvant	No	1	Anti-PD-1	No	38.8	289	26.1	26.1
8	Melanoma	79	90	Temporal	Adjuvant	No	1	Anti-CTLA4/ PD-1	Hypophysitis	29.6	53	6.5	6.5
