# Supplementary figures and images for: Five-Fraction Stereotactic Radiotherapy for Brain Metastases—A Retrospective Analysis
Source: Curr Oncol. 2023 Jan 17;30(2):1300–13. doi: 10.3390/curroncol30020101 (PMC9955428; doi:10.3390/curroncol30020101)

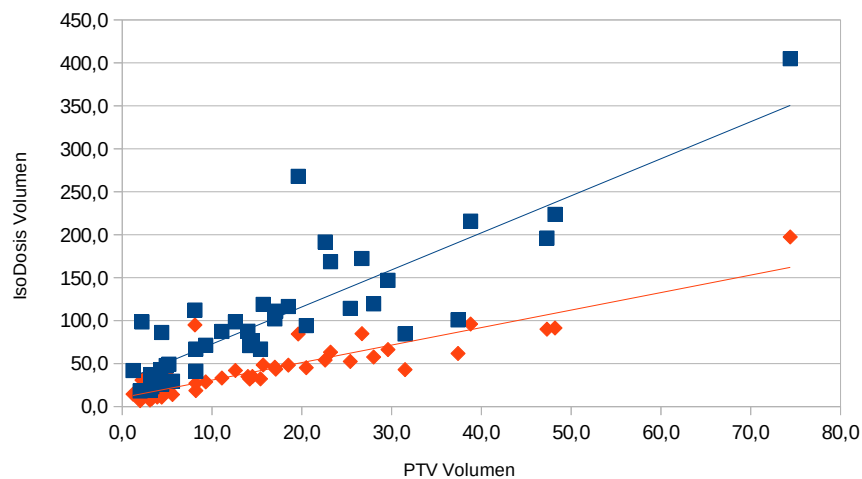

**A**

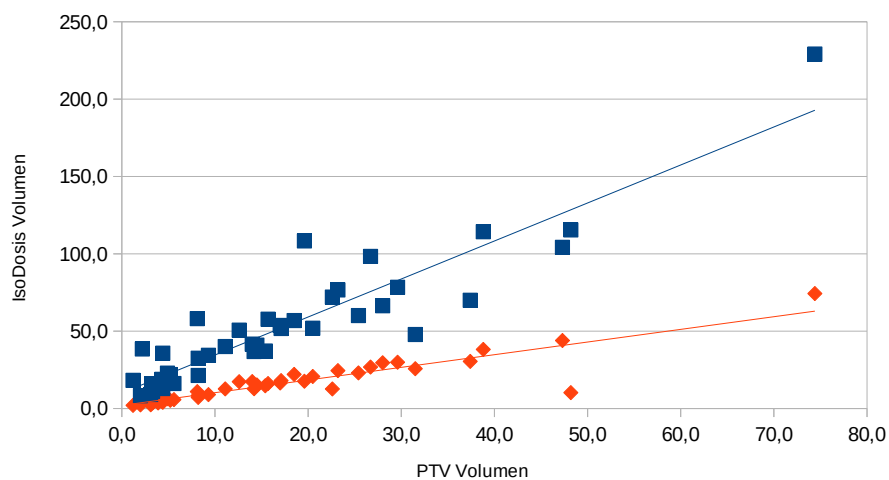

**B**

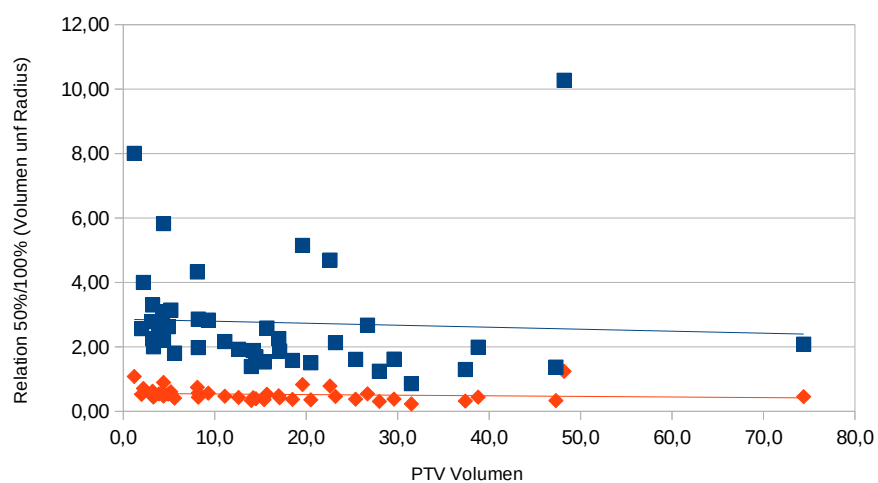

**C**

Supplement: Supplementary file 1 [file curroncol-30-00101-s001.zip › Supplementary Layer JP et al/Suppl. Figure S1.pdf]
